# Editorial of Special Issue “Current Trends in Chemistry Towards Biology”

**DOI:** 10.3390/ijms25137307

**Published:** 2024-07-03

**Authors:** Jiri Kos, Josef Jampilek

**Affiliations:** 1 Department of Biochemistry, Faculty of Medicine, Masaryk University, Kamenice 5, 625 00 Brno, Czech Republic; 2 Department of Analytical Chemistry, Faculty of Natural Sciences, Comenius University, Ilkovicova 6, 842 15 Bratislava, Slovakia; 3 Department of Chemical Biology, Faculty of Science, Palacky University Olomouc, Slechtitelu 27, 783 71 Olomouc, Czech Republic

One of the definitions of chemical biology is that it is a scientific discipline spanning the fields of chemistry, biology, and physics; it primarily involves the application of chemical techniques, tools, analyses, and often compounds (also known as chemical probes), which are produced through synthetic chemistry, in order to study and manipulate biological systems. A schematic overview of the main libraries/spaces of chemical biology is shown in Figure 1. An insight into the history of chemical biology is offered, among others, in the article by Morrison and Weiss [1]; however, the actual history of chemical biology is almost elusive [2]. In any case, chemical biology can be considered as an interdisciplinary field—a bridge between chemistry and biology, which uses molecules/probes and their binding to biological systems to investigate the structure of biological targets and the reactions/responses after they have been affected [3,4]. The concept of chemical biology can be applied both in the investigation of biological targets and compounds that can be used in influencing targets in plants/animals [5,6,7,8,9], as well as in the search for new compounds that can potentially be used as drugs in order to meet the concepts of druglikeness [10] and druggability [11].

Thus, chemical biology is very similar to the modern concept of medicinal chemistry [12], which has essentially been developing since the time of Paracelsus (Figure 2) [13,14] and from chemistry that was performed in apothecaries in the 18th and 19th centuries [15], as well as the investigation of structure–activity relationships based on biological testing in the 20th century [16] and modern drug research, which is driven not only by modern chemical approaches and computational design, but increasingly by pharmacology, clinical sciences, and the rapid onset of molecular biology and genomic sciences [17,18,19]; see Figure 3.

Therefore, it is not surprising that this Special Issue, entitled “Current Trends in Chemistry towards Biology”, contains articles covering the broad field of the discovery and investigation of bioactive compounds. A classical chemical biology and/or medicinal chemistry approach presents the design of the structural and biological features of G-quadruplex aptamers leading to them being promising antiproliferative compounds affecting the STAT3 signaling pathway [20]. The design of skin-protecting UV absorbers (excipients) is discussed by Yang et al. [21], while effective and stable lipase-based biocatalysts that are used in the synthesis of drug molecules are reported by Khiari et al. [22]. Modern approaches of the chemical analysis of biomaterials are represented by the studies in [23,24], while pharmaceutical analysis describing a new, simple, and universally applicable electrochemical detection method for paracetamol is discussed in [25]. The study in [26] deals with increasing the safety of drugs, while the toxicological study of propafenone, with its detailed tissue distribution, is described in [27].

From the above, chemical biology as a progressive, modern, multidisciplinary interface to solve the problem of drug discovery is not only gaining a lot of attention from scientists/scientific teams, but is also widely supported due to the expected achievements in understanding many diseases.

## Figures and Tables

**Figure 1:**
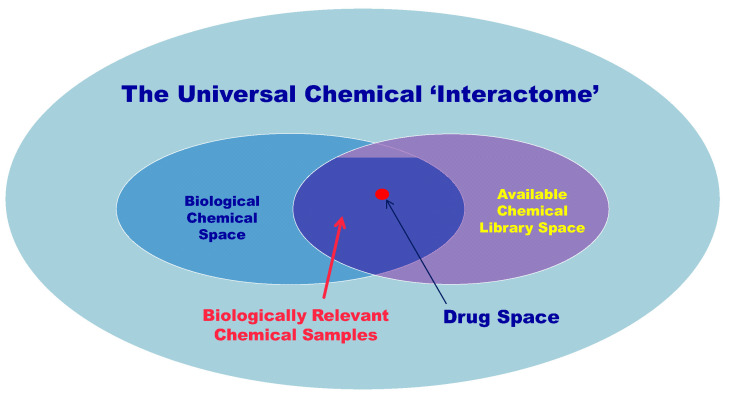
Interaction of biological chemical space with available chemical space.

**Figure 2:**
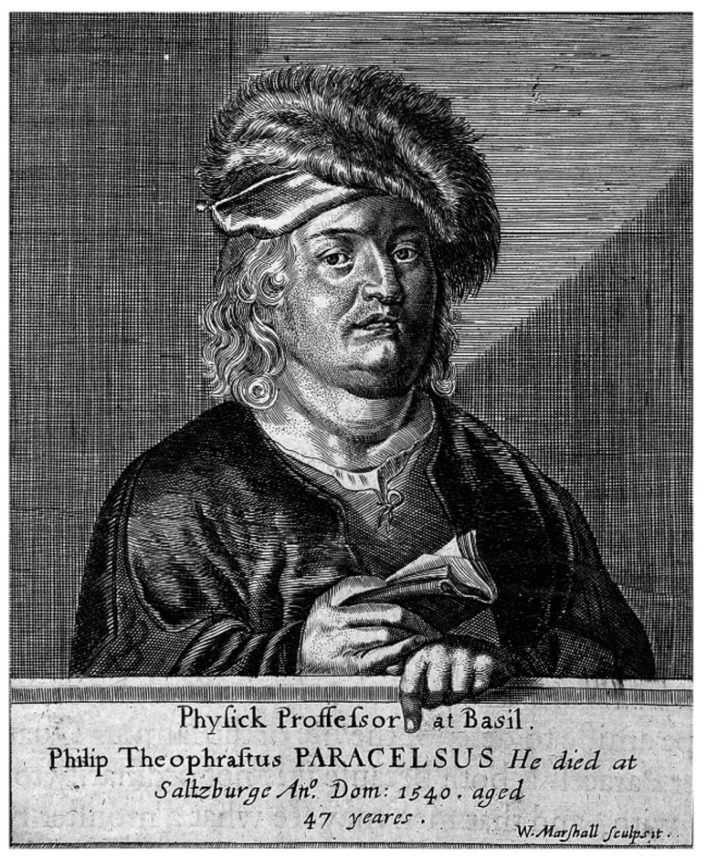
Theophrastus Bombastus Von Hohenheim known as Paracelsus. Line engraving by W. Marshall after J. Payne. Source: Wellcome Library London. Adapted from [14]. Copyright 2021 Elsevier B.V.

**Figure 3:**
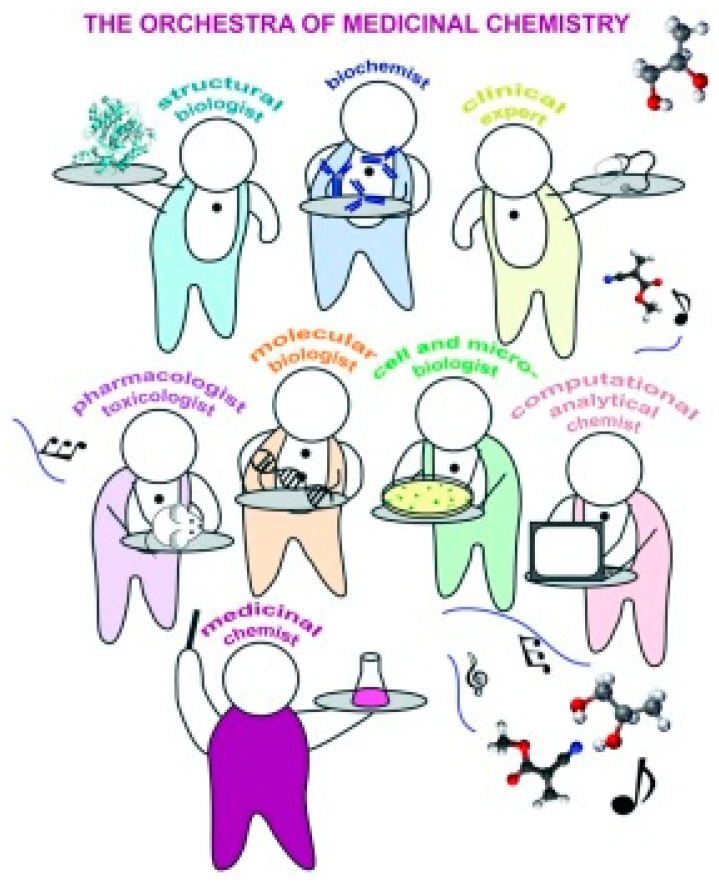
Cartoon showing how medicinal chemists orchestrate the discovery of new molecules to improve health by coordinating efforts amongst numerous disciplines. Adapted from [12]. Copyright 2017 The Royal Society of Chemistry.
